# Vitamin D Deficiency and Effects of Vitamin D Supplementation on Disease Severity in Patients with Atopic Dermatitis: A Systematic Review and Meta-Analysis in Adults and Children

**DOI:** 10.3390/nu11081854

**Published:** 2019-08-09

**Authors:** Sonal R. Hattangdi-Haridas, Susan A. Lanham-New, Wilfred Hing Sang Wong, Marco Hok Kung Ho, Andrea L. Darling

**Affiliations:** 1 Department of Nutritional Sciences, School of Biosciences and Medicine, Faculty of Health and Medical Sciences, University of Surrey, Guildford, Surrey GU2 7XH, UK; 2 Department of Paediatrics and Adolescent Medicine, Queen Mary Hospital, LKS Faculty of Medicine, The University of Hong Kong, Hong Kong, China

**Keywords:** 25-hydroxyvitamin D, 25(OH)D, systematic review, case-control, supplementation, meta-analysis, atopic dermatitis, paediatric, randomised control trial, SCORAD

## Abstract

Research has investigated 25-hydroxyvitamin D (25(OH)D) levels in the Atopic Dermatitis (AD) population, as well as changes in AD severity after vitamin D (VitD) supplementation. We performed an up-to-date systematic review and meta-analysis of these findings. Electronic searches of MEDLINE, EMBASE and COCHRANE up to February 2018 were performed. Observational studies comparing 25(OH)D between AD patients and controls, as well as trials documenting baseline serum 25(OH)D levels and clinical severity by either SCORAD/EASI scores, were included. Of the 1085 articles retrieved, sixteen were included. A meta-analysis of eleven studies of AD patients vs. healthy controls (HC) found a mean difference of −14 nmol/L (95% CI −25 to −2) for all studies and −16 nmol/L (95% CI −31 to −1) for the paediatric studies alone. A meta-analysis of three VitD supplementation trials found lower SCORAD by −11 points (95% CI −13 to −9, *p* < 0.00001). This surpasses the Minimal Clinical Important Difference for AD of 9.0 points (by 22%). There were greater improvements in trials lasting three months and the mean weighted dose of all trials was 1500–1600 IU/daily. Overall, the AD population, especially the paediatric subset, may be at high-risk for lower serum 25(OH)D. Supplementation with around 1600 IU/daily results in a clinically meaningful AD severity reduction.

## 1. Introduction

Atopic Dermatitis (AD) is a chronic recurrent inflammatory disease of the skin characterised by pruritus and inflamed lesions, involving specific areas of the body and causing generalised xerotic skin. As the disease progresses from acute to subacute, to chronic stages, excoriation from scratching and a propensity to secondary infections leads to oozing lesions and further pruritus. Severity of pruritis is associated with quality of life. Scores on the PO-SCORAD (patient oriented SCORAD) questionnaire, which includes a visual analogue scale for pruritis severity, are associated with measures of quality of life [1,2].

Eighty-five percent of AD is seen in children, of which 30% continue to suffer in their adult years [3]. There are an estimated 15 million suffers in the United Kingdom (UK) [4] and in the United States (US); 25% of children and 7% of adults have AD [3]. The incidence of AD is growing world-wide, especially in urbanised countries, with a higher rate in northern latitudes during the winter months [5]. The financial strain of AD at the level of individual, family and the public healthcare system cannot be underestimated. A 4.2 billion USD per year cost was estimated for the US alone with individual healthcare costs for AD patients higher by between 28.3% and 67.9% compared to non-AD patients [6,7].

AD pathology involves a complex interplay of barrier issues of skin and various dysfunctions in host innate and adaptive immune systems. These include high IgE, eosinophil and distinct T-helper cell populations as well as cytokine dysmodulation [8,9,10]. Bacterial and viral infestation from Staphylococcus Aureus and Herpes Simplex infections exacerbate pre-existing AD. However, factors contributing to long-term remission of AD are currently unknown [11,12]. Presently, due to no identified clinical biomarkers, quantitative and qualitative clinical tools are used to gauge severity of clinical presentation, with the Scoring Atopic Dermatitis index (SCORAD) being the most validated and commonly used in clinical research. The severity scale for SCORAD is pegged at Mild AD <25 points, Moderate AD >25 points and Severe AD >50 points [13,14].

There is much interest in the potential role of vitamin D deficiency in the development of AD, from multiple lines of research evidence. First, research has documented the aggravation of AD in winter, especially in higher latitude countries where serum 25(OH)D tends to be particularly low in this season [5]. Second, improvement in AD symptoms in patients has been observed in research studies on VitD supplementation [15,16]. Third, genetic polymorphisms including those of the Vitamin D Receptor (VDR) and a filaggrin gene mutation (up to 50% of the AD population, depending on specific mutation) have been identified as contributors to the development of AD [17,18].

Of note, Vitamin D_3_ (VitD_3_) is known to play a role in the skin barrier function, as it modulates structural proteins of the cornified dermis layer, regulating the glycoseramides essential for the hydrating protective lipid barrier which keeps the skin moisturized [19]. It modulates innate immunity via the production of the anti-microbial peptides (AMPs) cathelicidin and defensin which can help reduce skin infection risk [20]. In addition, Amon et al. (2018) discussed how vitamin D has inhibitory effects on monocyte production (via Toll-like receptors) and well as inhibiting dendritic cell activity and increasing mast cell release of IL10 [21]. They also discussed how vitamin D reduces the release of proinflammatory cytokines from Th1 cells and inhibits the release of IgE by reducing B cell function [21]. These mechanisms would theoretically aid the reduction of chronic inflammation in the skin.

The optimum 25(OH)D level for the prevention of, or rehabilitation of, inflammatory skin diseases is yet unknown. At present, for bone health, the US Endocrine Society recommends that serum 25(OH)D levels <50 nmol/L (20 ng/mL) are classified as deficient and 53–73 nmol/L (21–29 ng/mL) as insufficient. The UK Scientific Advisory Committee on Nutrition recommendations are more conservative, suggesting 25 nmol/L or higher as a population protective level for bone health [22]. These are population estimates and the physiological need for vitamin D may be higher in some clinical conditions. More research is now required to assess the optimum serum 25(OH)D concentration specifically required for those with AD, as well as assessing the current 25(OH)D status in AD patients.

Two previous systematic reviews and meta-analysis in 2016 on vitamin D and AD [12,23] have found a lower serum 25(OH)D in AD patients compared with non-AD patients [12] as well as a reduced severity of disease in AD patients after vitamin D supplementation [12,23]. However, new research has been conducted since the publication of these reviews and so there is a clear need for this systematic review and meta-analysis to be updated. A recent systematic review reviewed the area [24] but did not include a meta-analysis so there was no updated effect size using data from recent trials. The aim of this work therefore was to provide an updated review of observational and intervention trial data on the role of VitD in AD, including all published studies up to February 2018. First, we assessed the mean difference, in observational studies, between 25(OH)D concentration in AD patients and HC, with a sub-analysis of adult and paediatric populations separately. This was to gauge differences in 25(OH)D from normal which may be of clinical importance. Second, we assessed the impact of VitD supplementation on AD severity (change in SCORAD index), quantifying the role of VitD dosage and trial duration, to both support further research and advise clinical guidelines.

## 2. Methods

### 2.1. Search Procedures

A systematic search was conducted on the MEDLINE database via Pubmed (https://www.ncbi.nlm.nih.gov/pubmed/). The EMBASE database (https://www.embase.com/login) and the COCHRANE DATABASE for registered trials (http://cochranelibrary-wiley.com/cochranelibrary/search?searchRow.searchOptions.searchProducts=clinicalTrialsDoi) were also searched. For search terms, MESH i.e., Medical Subject Headings (MESH) were used along with pre-text terms. Search terms used for all three databases included: “Vitamin D AND Atopic Dermatitis”; “Vitamin D AND Eczema”; “25 Hydroxy Vit D AND Atopic Dermatitis”; “Vitamin D AND Atopic Dermatitis AND children”.

Following the recommendations by the Cochrane Database of Systematic Reviews (http://www.cochranelibrary.com/), reference lists searches were made to double-check for appropriate papers not previously located in the electronic searches. We also searched for grey literature such as abstracts from conference presentations. Researchers with abstract publications in the field were contacted via email and phone calls to request complete study results and data sets. Researchers of fully published papers were also contacted for any missing data. The searches covered papers from January 1963 to February 2018. In studies which included both adult and child participants, published data and raw unpublished data of both adult and child participants were obtained to enable sub-analysis for the adult and child populations. This review did not require ethical approval as it only involved analysis of already collected data.

### 2.2. Eligibility Criteria for Inclusion and Data Extraction

Observational studies (case-control design) as well as interventional studies, including randomized double-blind placebo control trials, non-randomized placebo control trials, clinical intervention and audit trials were assessed. We included all human studies published in the English language. Inclusion criteria were as follows: Age group >1 year, including both males and females. Exclusion criteria were as follows: Pregnant women, infants <1 year. Intervention studies were only included if they had an assessment of serum 25(OH)D levels at baseline. For both observational and intervention studies, the SCORAD and/or the EASI score needed to be included in the study for inclusion in the systematic review. This helped support clear mathematically calculable data in terms of proof of AD and severity of presence of AD in observational studies and improvement or exacerbation of AD during the interventional trials. For interventional studies, this enabled calculation of an effect size for the effect of VitD supplementation on AD severity, giving a quantifiable estimate to assess the clinical relevance of the results. Search procedures were documented using the PRISMA protocol.

### 2.3. Primary Outcomes

1. From observational studies, serum 25(OH)D levels in AD patients versus HC.
2. From interventional trials, changes in SCORAD or EASI score in the VitD supplemented AD group compared to the placebo supplemented AD group.

### 2.4. Secondary Outcomes

1. The relationship between serum 25(OH)D levels in AD with SCORAD or EASI score.
2. Effects of VitD supplementation on secondary infections of skin in AD.
3. The relationship between serum 25(OH)D levels, serum IgE levels and total eosinophil count (TEC) in the AD population and HC, including changes in these post-supplementation.
4. In interventional trials only: Effects of the co-usage of topical steroids.
5. The relationship, in AD patients, between serum 25(OH)D levels and cathelicidin LL-37 or Cathelicidin Antimicrobial Peptide (CAMP) and changes post-supplementation.
6. The relationship, in AD patients, between serum 25(OH)D levels and serum cytokines, with changes post-supplementation.
7. The relationship between serum 25(OH)D levels and atopic sensitisation.

### 2.5. Statistical Analysis

The *p*-value, confidence interval, and effect estimate of all primary outcomes were extracted. For observational studies, included in the meta-analysis of comparison of 25(OH)D levels in AD and HC, the mean ± standard deviation (SD) of 25(OH)D levels of both groups were extracted, as well as *n* (number of participants) in each group. Serum 25(OH)D concentrations were inputted into the meta-analyses in nmol/L. The serum 25(OH)D concentrations in two studies were presented in nmol/L [25,26]. The other nine studies [27,28,29,30,31,32,33,34,35] presented 25(OH)D concentrations in ng/mL but were converted to nmol/L using the standard formula: nmol/L = 2.5 ng/mL.

For interventional studies, as well as the number of participants (*n*) in the intervention group and the control group, the SCORAD score was extracted at baseline and after intervention (mean ± SD). In one study [36], the post intervention score was represented as a percentage of improvement so the mean ± SD were calculated accordingly for this study. For supplementation trials, VitD dose and trial duration were also extracted.

Review Manager (Rev Man 5.3; Cochrane Collaboration, London, UK) was used to perform the meta-analyses. The Newcastle–Ottawa Scale and the Cochrane Risk of Bias Scale were used to assess the quality of observational studies and interventional studies, respectively. The *I*^2^ statistic was used to assess heterogeneity between study outcomes and, due to significant between-study heterogeneity, the meta-analyses were conducted using the random effects model.

A planned a priori sub-analysis, for adult and paediatric specific data, was undertaken for the meta-analysis of serum 25(OH)D levels in AD and HC. Data from the two studies with data for separate age groups [31,32] were extracted from their published papers and additional raw data were also supplied by their research teams. The meta-analysis of interventional studies was undertaken using changes in SCORAD score between baseline and post-VitD supplementation, a weighted mean dose across this meta-analysis was calculated. This was calculated manually by multiplying each trial dose by the weighting given for that study in the meta-analysis and summing up the dosage to represent the 100% weighted mean dose. A planned a priori sub-analysis of interventional trials was done to assess the difference in SCORAD score changes in relation to VitD dosage and time period of trials. Inspection of funnel plots were undertaken to assess potential publication bias (subject to restrictions incurred by the number of studies available for analysis). Sensitivity analyses were also conducted when the analysis contained more than two studies.

## 3. Results

### 3.1. Systematic Review

**Figure 1:**
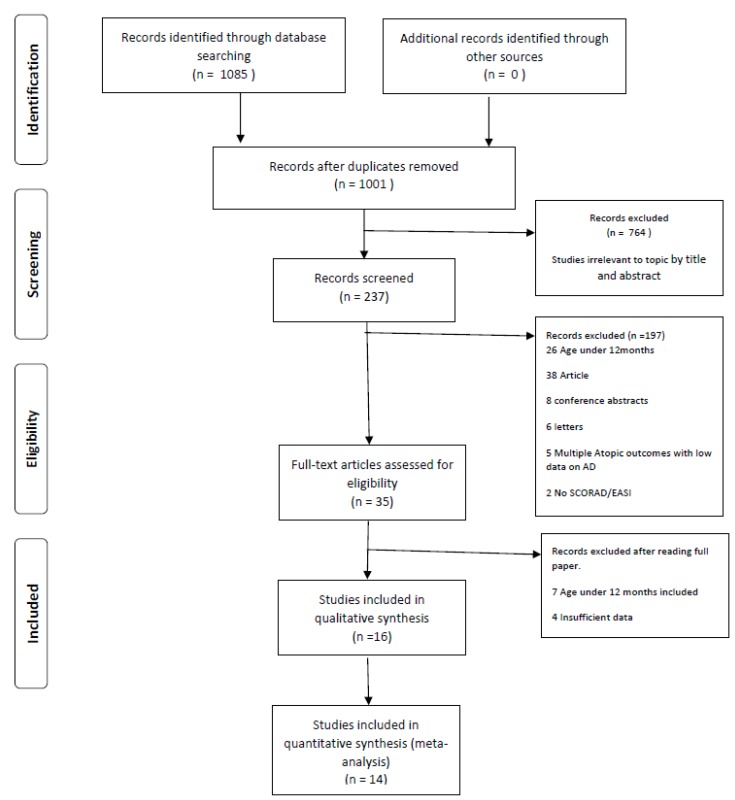
PRISMA 2009 Flow Diagram to show results of the search process and inclusions/exclusions.

Results of the systematic literature searches are shown in Figure 1. The textual Systematic Review assessing results for each outcome and its relation with 25(OH)D status can be viewed in the Supplemental File. Table 1, Table 2, Table 3 and Table 4 illustrate the characteristics and outcomes of the included studies. Results of the Newcastle–Ottawa Scale and the Cochrane Risk of Bias Scale scoring can be seen in Supplementary Tables S1 and S2.

### 3.2. Meta-Analysis

#### 3.2.1. Serum VitD in AD Compared to HC, with Sub-Analysis of the Paediatric Population

**Figure 2:**
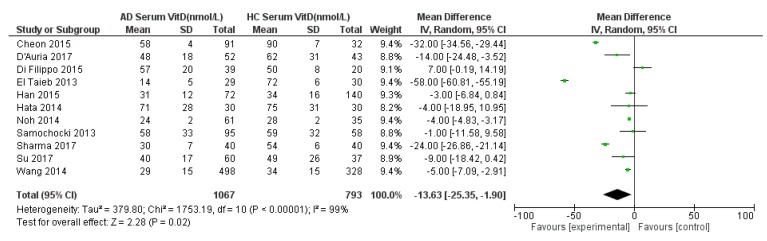
Forest Plot for meta-analysis of serum 25(OH)D levels in atopic dermatitis population compared with healthy controls (nmol/L). References: Cheon 2015 [27]. D’Auria 2017 [28]. Di Filippo 2015 [29]. El Taeib 2013 [30]. Han 2015 [31]. Hata 2014 [35]. Noh 2014 [32]. Samochocki 2013 [33]. Sharma 2017 [25]. Su 2017 [34]. Wang 2014 [26].

Eleven studies were suitable for inclusion in the meta-analysis to compare serum 25(OH)D in AD compared to HC (Figure 2). Three studied a mixed population of adult and paediatric populations [31,32,35]. One study [33] studied an adult only population whilst seven studies documented serum 25(OH)D levels in paediatric populations only [25,26,27,28,29,30,34]. Figure 2 summarizes the meta-analysis for serum 25(OH)D in AD and HC, including eleven studies. The results showed a statistically significantly lower 25(OH)D concentration in AD patients than HC by −14 nmol/L (95% CI −25 to −2, *p* = 0.02; *I*^2^ = 99%). Very high heterogeneity was noted so a random effects model was used.

A sensitivity analysis was performed (Table S3). The effect size stayed very stable when studies were removed in turn, except for being lower (around a 9 nmol/L group difference) when El Taieb et al. (2013) [30] was removed. Also, the removal of Cheon et al. (2015) [27], Wang et al. (2014) [26], and Sharma et al. (2017) [25] rendered the effect size not statistically significant. A further sensitivity analysis was done specifically for studies from common regions and ethnicities, including 3 studies from South Korea and two studies from Italy (Table S4). The effect size was not statistically significant when including only the Korean studies, although the magnitude of the effect was similar to when all studies were included (25(OH)D status was still around 14 nmol/L lower in the AD than in the non-AD group). The inclusion of only the Italian studies still gave a statistically significant result, with a similar effect size to that for all studies (16 nmol/L group difference).

**Figure 3:**
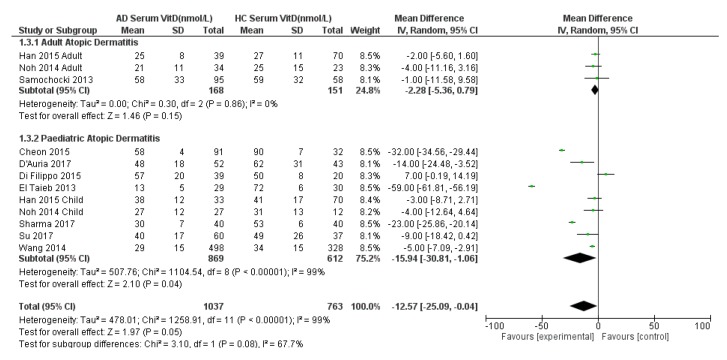
Forest Plot of comparison of serum 25(OH)D levels (nmol/L) in adult and paediatric atopic dermatitis populations versus their age-matched healthy controls, with sub-analysis by age group. References: Cheon 2015 [27]. D’Auria 2017 [28]. Di Filippo 2015 [29]. El Taeib 2013 [30]. Han 2015 [31]. Hata 2014 [35]. Noh 2014 [32]. Samochocki 2013 [33]. Sharma 2017 [25]. Su 2017 [34]. Wang 2014 [26].

A sub-analysis by age was also conducted, separating the purely adult AD and paediatric AD populations with their same age HC (Figure 3). The sub-analysis for the paediatric population showed a statistically significantly lower serum 25(OH)D in AD children by 16 nmol/L compared to HC: −16 (95% CI −31 to −1, *p* = 0.05, *I*^2^ = 99%). Considerable heterogeneity was noted (*I*^2^ = 99%) so a random effects model was used. The sub-analysis for the adult AD population did not show a statistically significant result: −2 nmol/L (95% CI −5 to 1, *p* = 0.15, *I*^2^ = 0%). The effect size for the adult population studies may need to be interpreted with caution due to presence of few included studies and a small population (*n* = 168 AD vs. *n* = 151 HC) due to limited data available.

Finally, the overall adult and child sub-analysis showed a statistically significantly lower 25(OH)D mean difference in AD patients by −13 nmol/L (95% CI −25 to −0.04, *p* = 0.08; test for subgroup difference *I*^2^ = 68%, *p* = 0.08) (Figure 3). Of note, this result differs slightly from Figure 2 due to the differential weighting of each study when split by age group and then the adult and child estimates are pooled compared with when there is no split by age group.

Funnel plots for the analyses represented in Figure 1 and Figure 2 were conducted (Supplementary Figures S1 and S2). Both plots showed asymmetry with relatively few studies with positive effect sizes (i.e., case serum 25(OH)D higher than control) suggesting possible publication bias.

#### 3.2.2. VitD Interventional Trials and Change in Clinical AD Severity (SCORAD)

**Figure 4:**
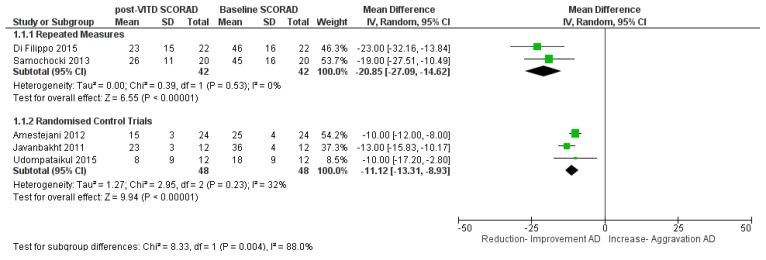
Meta-analysis of vitamin D intervention trials in atopic dermatitis: Comparison of clinical SCORAD index at baseline and post-vitamin D supplementation. References: Amestejani 2012 [39]. Di Filippo 2015 [29]. Javanbakht 2011 [40]. Samochocki 2013 [33]. Udompatailkul 2015 [36].

A meta-analysis was conducted for five interventional trials in AD cases with the primary outcome of change in SCORAD index after intervention compared to baseline (Figure 4). Two trials were from a combined adult and paediatric population [39,40]. One trial was from a purely adult population [33] while two trials were from paediatric AD populations [29,36]. The analysis was performed as two subgroups as the results from repeated measures interventions (i.e., patients are their own control) could not be statistically combined with studies that involved two groups of individuals (intervention vs. placebo).

For the repeated measures interventions, there was a highly statistically significant reduction in SCORAD by 21 points on intervention with VitD, (mean difference = −21 points (95% CI −27 to −15, *p* < 0.0001; *I*^2^ = 0%) with a weighted mean dose of 1500 IU/daily. For the randomised control trials, there was a highly statistically significant reduction in SCORAD by 11 points on intervention with VitD, (mean difference = −11 points (95% CI −13 to −9, *p* < 0.0001; I = 32%) with a weighted mean dose of 1600 IU/daily (Figure 4). The random effects model was used for the analyses as substantial heterogeneity was seen between studies for the randomised control trials. A sensitivity analysis was performed (Table S5), removal of each study in turn only had a small effect on effect size and no effect on statistical significance.

Though the number of studies were few, all studies showed improvement in SCORAD index on supplementation with VitD. It should be noted that in these five studies the AD population consisted mostly of mild and moderate AD with few severe cases in two of the studies.

A sub-analysis by dosage and duration for the five trials (Supplementary Figure S3) showed a greater change in Di Filippo et al. (2015) [29] and Samochocki et al. (2013) [33], which were both of three month duration and repeated measures, compared with the other trials which were randomised control trials and only one to two months duration. In the paediatric Di Filippo et al. (2015) [29] study, VitD dosage was 1000 IU/daily compared with 2000 IU/daily in the adult Samochocki et al. (2013) [33] trial. This sub-analysis must be interpreted with caution due to the limited number of studies, with substantial heterogeneity. Overall, it is noted that the three-month trials contained mostly mild–moderate AD patients, were repeated measures studies rather than randomised control trials and showed higher mean reduction in SCORAD than the one to two month studies.

## 4. Discussion

Our findings show a lower serum 25(OH)D concentration by 14 nmol/L in the overall adult and paediatric AD population than in HC, with lower serum 25(OH)D also in the AD paediatric population by 16 nmol/L. There was no difference in the adult population alone, the effect size for which did not reach statistical significance. Therefore, our study shows that the AD population have lower 25(OH)D concentration than their healthy peers, particularly for children. Our results suggest that the AD paediatric population may be an “at-risk group” for VitD insufficiency. As per US Endocrine Society guidelines, all individuals at risk of vitD insufficiency must be assessed routinely for 25(OH)D status [37]. This should be considered as best practice during the diagnosis and treatment of the AD paediatric population. VitD supplementation may be considered by the clinician taking into account baseline 25(OH)D status and possible contraindications (e.g., endocrine dysfunction).

Pooling results from repeated measures clinical trials in AD patients, post-supplementation we found a highly statistically significant difference between supplement and placebo groups in SCORAD of −21 points, using dosages of 1000–2000 IU daily for three months. Similarly, pooling results from VitD randomised control trials in AD patients, post-supplementation we found a highly statistically significant difference between supplement and placebo groups in SCORAD of −11 points, using dosages of 1000–2000 IU daily for one to two months.

In intervention trials, the minimal difference or improvement set as a measure of effectivity of the intervention is called the Minimal Clinical Important Difference or MCID. For the treatment of atopic dermatitis, the MCID of the SCORAD score which translates to clinical relevance is a reduction of 9 points [42]. Our effect size of −11 to 21 points exceeds this threshold, suggesting clinical relevance. Therefore, we have found clear evidence for a clinically meaningful reduction in AD disease severity after VitD supplementation. Of note, the baseline average (mean or median) 25(OH)D for all intervention trials in the meta-analysis was <50 nmol/L, which would be classified as deficient [33,37]. This shows that the trials included individuals who were truly deficient in 25(OH)D at baseline, so were not (on average) supplementing individuals who already had sufficient 25(OH)D status. After supplementation, this average 25(OH)D was >50 nmol/L in all trials except one [33].

Bearing in mind the clinical background of the each patient, including individual 25(OH)D concentration and any existing endocrine issues, this research supports the empirical supplementation of daily VitD doses of approximately 1500–1600 IU/daily to AD patients, taking into account the baseline vitamin D levels and eventual endocrine issues or other concomitant diseases that contraindicate vitamin D supplementation.

This weighted mean of 1500–1600 IU (38–40 micrograms) daily dosage falls well below the European Food Standards Agency (EFSA) Tolerable Upper Intake Levels (UL) of 100 micrograms per day for all adults as well as children aged 11–17 years [43]. It also falls below the UL for 1–10 year old children (50 micrograms per day) [43] but is higher than the UL for infants (25 micrograms per day) [43]. Clinical biochemical monitoring should be undertaken of children receiving 1500–1600 IU (38–40 micrograms per day) due to it being closer to their UL (50 micrograms per day) and infants should definitely not be given 1500–1600 IU/daily. Indeed, the UL is not a target, and is based on population (not individual) safety. Clinicians should make their own judgements about safe intakes for their individual patients, bearing in mind the effective dose suggested by this study (1500–1600 IU/daily).

In our analysis, larger results were seen in trials of three month duration but from the limited data available we were not able to assess whether this was due to the 3 month duration itself, or if it was simply that all the three month studies were repeated measures studies, rather than full randomised placebo control trials. Nevertheless, it stands to logical reason that supplementing for three months, rather than one to two months, is likely to lead to a better clinical response.

In terms of previous systematic reviews and meta-analyses in the field, our work supports the findings of systematic reviews by Kim et al. (2016) [12], Kim and Bae (2016) [23], Huang et al. (2018) [24] and Vaughn et al. (2019) [44] in terms of finding a lower 25(OH)D status in AD patients than controls, and finding an effect of vitamin D supplementation on symptom severity.

Our textual systematic review indicated that most interventional trials have documented a reduction in skin infection after VitD supplementation. Some observational evidence also suggested an association between lower 25(OH)D concentration and increased cutaneous secondary-colonisation of *S. aureus* and herpes, suggesting that increasing 25(OH)D levels in the AD population may support the reduction of and prevention of secondary cutaneous infections, albeit this was based on a small number of studies and there was not enough data to perform a meta-analysis.

In terms of biological mechanisms, it is feasible that VitD could affect the severity of AD, including number of infections. VitD is known to modulate innate and adaptive immune responses [45]. The physiological role of VitD in supporting healthy skin [20], as well as the fact that lower 25(OH)D concentrations are known to correlate with increased allergic sensitisation [46], higher IgE level [47], and lower serum cathelicidin levels [48], suggest a role of VitD in modulating AD severity. Moreover, studies involving the disruption of the VDR have showed lower levels of involucrin, profilaggrin and loricin barrier proteins [49]. Improvement in 25(OH)D leads to upregulation of functional human cathelicidin (hCAP18) in keratinocytes from AD patients, as well as from those from patients with psoriasis and normal skin [50]. In support of the above mechanisms, Albenali et al. (2016) [38] showed that higher IgE levels, higher virulence and colonisation of *S. aureus* were recorded when serum 25(OH)D levels were low. A significantly increased risk of having skin lesions with methicillin-resistant *S. aureus* (MRSA) has been found in persons with VitD deficiency [51,52,53]. Udompataikul et al. (2015) [36] found a reduction in *S. aureus* colonization in a paediatric population on VitD supplementation while Samochocki et al. (2013) [33] found no incidence of infection in their adult supplemented population. Albenali et al. (2016) [38] observed a 4-fold upregulation of LL-37 in the stratum corneum on VitD supplementation and a reduction in AD complicated by eczema herpeticum.

Secondary infections and re-infections in AD are notoriously challenging to treat, with excess use of topical and oral antibiotics increasing the risk of microbial antibiotic resistance. A recently published study [54] analysed eleven-year nationally representative data and calculated the morbidity, mortality and cost of secondary infections in AD to be in excess of 11 to 228 million USD annually. Improving 25(OH)D levels in AD may support the war on antibiotic resistance by reducing the risk and severity of cutaneous infections. However, a lot more research is needed on this subject due to the small amount of currently published literature.

In terms of strengths, our systematic review and meta-analysis is the most up to date available on the role of VitD in AD in both adults and children. It calculated pooled effect sizes in terms of mean difference in serum 25(OH)D levels between the AD population and HC. Our effect size is also larger than the mean difference found in the other meta-analysis by Kim and Bae (2016) [23]. Our review is the first to document clinically relevant changes in disease severity (as assessed by SCORAD) after VitD supplementation.

In terms of limitations, it is important to note that the data from trials in our analysis included mainly mild and moderate AD with only a few severe cases. Also, no data from infants (<1 year of age) or pregnant women were included. The specific reduction in SCORAD seen, and difference in 25(OH)D between AD patients and HC may differ in these groups from that found in this review. Finally, our results are based on a mean weighted dose of around 1500–1600 IU per day and SCORAD reductions observed seen are likely to differ with higher or lower doses.

Three trials [35,38,41] could not be included in the meta-analysis due to no reporting of the standard deviation for SCORAD. These happened to be the higher dose trials and so this limited our analysis to trials with dosage ranges of 1000 IU–2000 IU/daily. Six trials confirmed the form of VitD given as VitD_3_ or VitD_2_, but two trials did not report the form of VitD used. The longest trials were only of three months duration hence the effect of longer-term supplementation could not be analysed. Our meta-analysis of 25(OH)D concentration in AD compared to HC was limited by the small sample size, especially for the adult population. Similarly, the meta-analysis of the interventional studies was limited by the small number of trials suitable for inclusion.

Quality analysis of the interventional studies showed four higher scoring randomised double blind clinical trials with mention of adequate randomisation and blinding [35,36,39,40]. In terms of the other studies, one study was designed as a clinical evaluation study and so was not a randomised clinical trial, [38] and Samochocki et al. (2013) [33] did not mention randomisation but confirmed blinding of both participants and researchers. Di Filippo et al. (2015) [29], Albenali et al. (2016) [38] and Tsotra et al. (2017) [41] did not mention randomisation or blinding of participants.

There was some potential evidence of publication bias in that there was asymmetry in the funnel plots, with very few studies having positive effect sizes (i.e., cases having higher serum 25(OH)D than controls). However, the funnel plots only contained a small number of studies (*n* = 11 or *n* = 12) so they must be interpreted with caution. The number of interventional studies were too few to judge publication bias so there may still be a possibility of unpublished studies with null findings, despite best efforts being made to locate unpublished data. In some trials, the limited information on form of VitD supplemented (D_2_ vs. D_3_) and the absence of information on ingredient type and source of the D_2_ or D_3_ prevented further analysis in this regard.

In terms of further research, there is an urgent need for longer term, well conducted trials in distinct age categories, at different severity levels and also for different histopathological disease stages. Intervention trials with VitD dosage titration based on the severity of the disease, concomitant cytokines, the cell landscape and dermal cathelicidin levels are also needed. Trials designed to understand the link between VitD supplementation, skin barrier function and innate immunity to reduce secondary cutaneous infections in AD would help provide evidence that may justify the need for VitD supplementation to modulate the prevalence of microbe colonisation and reduce the need for antibiotics in these patients. Studies assessing effects of VitD supplementation on topical steroid usage would also be useful as reduced usage of steroids would have cost benefits. If optimal serum 25(OH)D levels could indirectly support the reduction of antibiotic usage and curtail antibiotic resistance, further research in this area is clearly urgently required. Particularly, research investigating and quantifying the effect of VitD on gut and skin microbiota may support supplementation as a possible preventative and adjuvant treatment strategy.

Further trials with specifically vitamin D_3_ vs. D_2_ may provide data to support form dosage and time period of the supplementation for fastest recovery with least risk. Assessing the source and ingredients in vitamin D supplements used in trials may shed new light on a population known for sensitisation especially in the younger years. VitD_3_ forms of supplements are likely to support a more efficient increase in serum 25(OH) levels [55] but are usually derived from lanolin (from sheep’s wool).

Finally, the textual systematic review suggested that studies of VitD supplementation in mild to moderate AD did not show changes in pruritus (based on SCORAD), skin xerosis, lichenification, skin conductance and moisture levels of skin [36]. This suggests the need for investigating other possible treatment strategies including, possibly targeting specific cytokines, such as monoclonal antibodies against IL-31 to reduce pruritus, as well as the use of nutritional factors, to support AD therapy.

## 5. Conclusions

Our study shows significantly lower 25(OH)D levels in the AD population, especially the paediatric subset. Monitoring of 25(OH)D levels in AD patients is warranted, especially in children. VitD supplementation trials showed clinically relevant improvements at a weighted average dose of 1500–1600 IU for up to 3 months. Clinicians should consider appropriate supplementation after evaluating patients’ 25(OH)D concentration and medical history. Further research is required to establish the efficacy of vitamin D_2_ vs. D_3_ in reducing AD severity, as well as the effects of VitD supplementation on infection rates, including superinfections and topical steroid usage.

## Figures and Tables

**Table 1:** Observational case-control studies of serum 25(OH)D levels in atopic dermatitis individuals compared to healthy controls.

Case Control Study	Participants	Population-Total *N*, ‘*n*-AD’, *n*-HC	Primary Study Outcome	Review Outcome Serum 25(OH)D Levels Observed	*p* Value	Secondary Study Outcome
Cheon 2015 (South Korea) [27]	Paediatric OPD, median age 6 years	*N*: 123, *n*-AD: 91, *n*-HC: 32	Serum 25(OH)D levels significantly lower in AD compared to HC. Lower levels in Moderate and Severe AD compared to Mild AD.	AD = 23 ± 2 ng/mL HC = 36 ± 3 ng/mL	<0.05	
D’Auria 2017 (Italy) [28]	Paediatric OPD. Age 1–14 years, 43% Caucasians, skin phototype II or III according to Fitzpatrick skin type	*N*: 95, analysis-*n*-AD: 52, *n*-HC 43	Serum 25(OH)D levels statistically significant higher in HC than AD even after adjustment for age, sex and season (*p* = 0.04)	AD = 19 ng/mL, HC = 25 ng/mL	0.04	No association was found between serum 25(OH)D levels and AD severity.
El Taieb 2013 (Egypt) [30]	Patients from the OPD Clinic. Age 2–12 years	*N*: 59, *n*-AD: 29, *n*-HC: 30	Mean Value of Serum Vitamin D in AD is much lower than HC.	AD = 5 ± 2 ng/mL, HC = 29 ± 2 ng/mL	<0.001	Mean Serum 25(OH)D levels significantly higher in Mild AD (15 ± 4 ng/mL) vs. Moderate AD (6 ± 3 ng/mL) or Severe AD (0.3 ± 0.1 ng/mL). Individual SCORAD values showed significant inverse correlation with serum 25(OH) D Levels, *r* = −0.88, *p* = 0.001
Han 2015 [31]	Patients: adult >18 years, child <18. Age: Adults: 26.8 ± 8.25 (18–51), Child 9.5 ± 4.27 (1–16) years	*N*: 212, Adults *n*-AD: 39, *n*-HC: 70, Children *n*-AD: 33, *n*-HC: 70	Serum 25(OH)D level significantly lower in AD children, not statistically different in AD adults. Overall not statistically different between 72 AD patients (12.43 ± 4.66 ng/mL) vs. 140 control (13.49 ± 6.23 ng/mL) (*p* = 0.05) All adults + 76% children with AD showed deficient levels of Serum 25(OH)D levels.	Child-AD = 15 ± 5 ng/mL, Child-HC = 16 ± 7 ng/mL. Adults-AD = 10 ± 4 ng/mL. Adult-HC = 11 ± 4 ng/mL	Child 0.04	Difference in serum 25(OH)D levels of different AD severity not statistically different (*p* > 0.05). Significant inverse correlation between BMI and VitD level in AD (*r* = −0.32, *p* = 0.007) and HC (*r* = −0.335, *p* = 0.009). Significant inverse correlation between SCORAD and serum LL-37 (*r* = −0.3, *p* = 0.01) for total population and only significant in adults after subdividing (*r* = −0.36, *p* = 0.025)
Noh 2014 (South Korea) [32]	Patients AD-82, Asthma-38 HC-49	*N*: 169, *n*-AD: 82, *n*-HC: 49. Analysis done with *n*-AD: 61, *n*-HC: 34	AD patients had significantly lower Vitamin D levels compared to Asthmatic pts and healthy Controls (*p* = 0.01 and *p* < 0.001). Statistically significant negative correlation between Serum 25(OH)D levels and eczema involvement of the total area (*r* = −0.376, *p* = 0.001)	HC = 11 ± 1 ng/mL AD = 10 ± 1 ng/mL	0.001	Significant inverse correlation was observed for serum 25(OH)D levels and total body affected by eczema (*r* = −0.38, *p* = 0.001) Correlation found between serum 25(OH)D levels and different dermal area manifestations, age, eosinophil count, serum IgE levels.
Sharma 2017 (India) [25]	Patients from Dermatological OPD, ages 2–18 years	*N*: 80, *n*-AD: 40, *n*-HC: 40	Lower Serum 25(OH)D levels in AD was statistically highly significant compared to HC	AD = 30 nmol/L, HC = 54 nmol/L	<0.001	Significantly inverse correlation between Serum25(OH)D levels and SCORAD index. Mean serum 25(OH)D levels were significantly lower in AD vs. HC with superficial bacterial, fungal, viral infection. Levels were deficient in patients with AD (<20 nmol/L) and insufficient in (20–29 nmol/L) in HC with cutaneous infection.
Su 2017 (Turkey) [34]	Dermatology OPD, 2–16 years (mean 8·37);	*N*: 97, *n*-AD:60, *n*-HC: 37	No statistical significant difference in Mean Serum 25(OH)D levels of AD and HC.	AD = 16 ± 7 ng/mL HC = 20 ± 10 ng/mL	0.07	Significant inverse relationship between SCORAD scores and Serum 25(OH)D levels. Serum 25(OH)D levels statistically significantly lower in moderate and severe AD compared with mild AD (*p* = 0.001, *p* = 0.004) Vit D showed negative correlation with Serum IgE levels (*p* = 0.007)
Wang 2014 (Hong Kong) [26]	Paediatric OPD (*n* = 405) Secondary school epidemiological study for obesity and diabetics (*n* = 95)	*N*: 826, *n*-AD: 498, *n*-HC:328	serum 25(OH)D levels higher in HC than AD. Inverse association seen between Serum VitD levels and SCORAD, Ness Scores.	AD = 29 ± 15 HC = 34 ± 15 nM	<0.001	Higher percentage of AD had Serum 25(OH)D <25 nM vs. HC (47.8% vs. 26.6%). Serum 25(OH)D levels showed inverse associations with short-term, long-term AD severity, serum IgE and eosinophil levels.

**Table 2:** Showing data of serum 25(0H)D levels in the atopic dermatitis participants compared to healthy controls in included studies.

Study	Case Study Type	n-Cases	n-HC	Age: Adult ‘A’ Child ‘C’	Serum 25(OH)VitD Levels AD Baseline (ng/mL)	Serum 25(OH)VitD Levels AD Baseline (nmol/L)	Serum 25(OH)VitD Levels Healthy Controls (ng/mL)	Serum 25(OH)VitD Levels Healthy Controls (nmol/L)	*p* Value	Geographical Location by Latitude	VitD Deficient(D) Sufficient(S) Insufficient(I)
Cheon 2015 [27]	Case-Control	91	32	C	23 ± 2	58 ± 4	36 ± 3	90 ± 7	<0.05	Korea 37.7	AD-I Controls-S
D’Auria 2017 [28]	Case-Control	52	43	C	19 ± 7	48 ± 18	25 ± 13	62 ± 31	0.04	Milan, Italy 45.5	AD-D Controls-I
Di Filippo 2015 [29]	Interventional	39	20	C	23 ± 8	57 ± 20	20 ± 3	50 ± 8	Not Specified	Cheiti, Italy 42.4	AD-I Controls-I
El Taieb 2013 [30]	Case-Control	29	30	C	5 ± 2	14 ± 5	29 ± 2	72 ± 6	<0.001	Egypt 26.8	AD-D Controls-I
Han 2015 [31]	Case-Control	72	140	A,C	12 ± 5	31 ± 12	14 ± 6	34 ± 16	*p* > 0.05	Korea 37.7	AD-D Controls-D
Han 2015 Adult [31]	Case-Control	39	70	A	10 ± 3	26 ± 8	11 ± 4	27 ± 11	*p* > 0.05	Korea 37.7	AD-D Controls-D
Han 2015 Child [31]	Case-Control	33	70	C	15 ± 5	38 ± 11·58	16 ± 7	41 ± 17	0.04	Korea 37.7	AD-D Controls- D
Hata 2014 [35]	Interventional	30	30	A,C	28 ± 11	71 ± 28	30 ± 12	75 ± 31	Not specified	Multicentric 32.7–45.5	AD-I Controls- S
Noh 2014 [32]	Case-Control	82	49	A,C	10 ± 0.6	24 ± 2	11 ± 1	28 ± 2	0.001	Korea 37.	AD-D Controls-D
Noh 2014—Child [32]	Case-Control	27	12	C	11 ± 5	27 ± 12	13 ± 5	31 ± 14	Retrieved from supplied data	Korea 37.7	AD-D Controls-D
Noh 2014 Adult [32]	Case-Control	34	23	A	9 ± 4	21 ± 11	10 ± 6	25 ± 15	Retrieved from supplied data	Korea 37.7	AD-D Controls-D
Samochocki 2013 [33]	Interventional	95	58	A	23 ± 13	58 ± 33	24 ± 13	59 ± 32	>0.05	Poland 51.9	AD-D Controls-D
Sharma 2017 [25]	Case-Control	40	40	C	12 ± 3	30 ± 7	21 ± 3	54 ± 6	<0.001	Punjab, India 31.2	AD-D Controls-I
Su 2017 [34]	Case-Control	60	37	C	16 ± 7	40 ± 7	20 ± 10	49 ± 26	0.07	Turkey 39	AD-D Controls-D
Wang 2014 [26]	Case-Control	498	328	C	11 ± 6	29 ± 15	14 ± 6	34 ± 15	<0.001	Hong Kong 22.4	AD-D Controls-D

Vitamin D status defined as a 25(OH)D—Deficiency <20 ng/mL (50 nmol/), Insufficiency 21–29 ng/mL (52.5–72.5 nmol/). Sufficiency >30 ng/mL (75 nmol/) [37].

**Table 3:** Interventional studies of vitamin D supplementation in atopic dermatitis.

Study	Interventional Design Type	Participants	Population-*n*	Primary Study Outcome	*p* Value	Secondary Outcome	Serum 25(OH)D Levels Baseline (nmol/L)	VitD Deficient(D) Sufficient(S) Insufficient(I)
Albenali 2016 (U.K.) [38]	Clinical service evaluation of AD and Eczema Herpeticum (ADEH). VitD supplementation based on deficiency and additionally 6000 IU/daily for age 1–12 years, 10,000 IU/daily for age 12–18 years. Baseline population: 57% with VitD def, 26% suboptimal levels and 83% insufficient VitD level	Age 1–18 years	*n* = 47, *n*-AD = 12, *n*-ADEH = 35	25(OH)D level and SCORAD showed significant inverse relationship (*p* = 0.01) *r* = −0.36. at baseline. After 2 months VitD supplementation, 42% reduction in SCORAD (*p* = 0.001)	0.001	Serum of AD was significantly correlated with LL-37 levels (*r* = −0.32 *p* = 0.01) suggesting causal relationship. LL 37 levels (known to help with re-epithelization) significantly different; reduced in severe AD, post-supplementation.	-	-
Amestejani 2012 (Iran) [39]	Randomized double-blind placebo controlled trial, 1600 IU/daily cholecalciferol given for 60 days	Age 14 and older.	Population *n* = 60 *n*-VitD = 24 *n*-placebo = 30	Significant improvement of SCORAD and TIS (Three Item Severity Score) value under in VitD group of mild, moderate and severe AD (*p* < 0.005) not seen in placebo group (*p* > 0.05).	<0.005		23 ± 3	D
Di Filippo 2015 (Italy) [29]	Interventional study—prospective longitudinal 1000 IU/daily 3 months.	Age 4 ± 3.15 year with AD and pre-pubertal Tanner stage 1.	*n*-AD = 39, final compliers AD = 22, *n*-HC = 20	Standard mean value of serum Vit D levels was insufficient and comparable with healthy controls. AD = 23 ± 8 HC = 20 ± 3. Improvement from baseline levels of serum 25(OH)D correlated with reduction in SCORAD index. Significant negative correlation between VitD change and SCORAD change (*r* = −0.49 *p* = 0.02)	0.01	In AD, high rate of VitD insufficiency (74%) and deficiency (7%). Improvement in VitD levels after 3 months correlated with reduction in SCORAD and reduced inflammatory cytokines.	57 ± 20	I
Hata 2014 (U.S.A-Multicenter) [35]	Randomized double-blinded placebo controlled trial VitD3: 4000 IU/daily for 21 days	AD interventional = 30 (mean age of 31.2 year); placebo = 30 (mean age 31.9 year)		AD subject with Fitzpatrick Type V/VI skin had significantly lower serum 25(OH)D level 19 ng/mL comparing to type III/IV with a mean 25 OHD of 29 ng/mL (*p* = 0.04). Increase in serum 25(OH)D from mean of 30 ng/mL to 40 ng/mL. *p* < 0.01	0.05	AD subject serum 25(OH)D inversely correlated with BMI (*r* = −0.31. *p* = 0.04).	29 (11)	D
Javanbakht 2011 (Iran) [40]	Randomized double-blind placebo controlled trial. 1600 IU/daily for 60 days	Age 13–45, SCORAD 10–70,	*n*-AD with VitD = 12, *n*-placebo = 11	SCORAD: significant reduction of 34.8% in VitD group, placebo group: 28.9%. Change in objective SCORAD: VitD group: 38%, placebo group: 31%. Change in intensity 37% VitD group, 25% in placebo group.	0.004	No associate relationship with SCORAD and serum 25(OH)VitD3 level. Strong reduction in usage of topical steroid.	-	-
Samochocki 2013 (Poland) [33]	Interventional study from a cross-sectional study not randomised or controlled. Blinding done for supplementation and SCORAD evaluation. 2000 IU/daily 3 months, Jan to March	Age 18–50 years	*n*-AD:95, *n*-HC:58 observational, For interventional study *n* = 20	Supplementation improved Vit D levels from deficient to insufficient levels, statistically significant decrease in AD severity (*p* < 0.05). All SCORAD parameters except lichenification significantly decreased. Correlation of improvement in SCORAD and VitD (*p* = 0.001)	<0.05	After 3 months supplementation mean total IgE level significantly lower than before (995 ± 1681 vs. 1148 ± 19 IU/mL) *p* < 0.001.	19 ± 8	D
Tsotra 2017 (Greece) [41]	Interventional study 2 months, Vitamin D supplementation 1200 IU in mild AD (SCORAD < 40), 2400 IU in severe AD (SCORAD > 40)	Children with AD, Severe and mild (no other information)	*n*-AD: 50,	Baseline SCORAD differed significantly between mild AD, severe AD group, *p* < 0.001. Post 20 days supplementation, the difference disappeared (*p* = 0.65) and statistically insignificant at 45 days (*p* = 0.61) and 60 days (*p* = 0.48). Significant “downregulation” of symptom severity in severe AD group on 2400 IU/daily oral VitD supplementation.	0.001	Levels of serum cathelicidin child with AD significantly higher than those with children in control group. AD 61 (261–129) *p* = 0.02, HC 50 (0.2–93.6). No correlation seen with severity of SCORAD and baseline serum cathelicidin and cathelicidin in tissue preparation for atopic skin.	-	-
Udompataikul 2015 (Thailand) [36]	Randomized double-blind placebo controlled trial VitD 2000 IU/daily.	1–18 years old. Mean age 8–28 years	*n*-Vit D: 12, *n*-placebo: 12	Changes of VitD group vs. placebo group at week 4: Statistically significant reduction in SCORAD (*p* = 0.02), lower *S. aureus* colony count (*p* = 0.03). Statistically significant reduction in Erythema index (*p* = 0.01). Oedema, excoriation, pruritus better. lichenification, dryness and sleeplessness not better. Skin conductance not statistically significantly difference between group at each visit (*p* = 0.01, 0.65 and 0.55 respectively) Serum VitD levels inversely correlate with SCORAD and *S. aureus* colonisation with statistically significant difference (*r* = −1.0, *p* = < 0.01). Obvious increment of 25(OH)D levels in supplemented group compared to placebo group significant difference 8 mg/mL vs. 0.8 mg/mL (*p* = 0.01)	0.02	Significant reduction documented in *S. aureus* colonisation in Vitamin D group at week 4 vs. placebo group, *p* = 0.03	43	D

Vitamin D status defined as a 25(OH)D—Deficiency < 20 ng/mL (50 nmol/L), Insufficiency 21–29 ng/mL (52. 5–72. 5 nmol/L). Sufficiency >30 ng/mL (75 nmol/L) [37].

**Table 4:** Data of interventional studies included in the meta-analysis.

Study	Age: Adult ‘A’ Child ‘C’	n-Cases	n-Controls	Serum 25(OH)D Levels Baseline(n/)	Serum 25(OH)D Levels after VitD Intervention (n/)	SCORAD before Intervention	SCORAD after Intervention	Percentage Improvement of SCORAD	*p* Value	Trial Period in Months	VitD Dosage IU.	Percentage Weight of Study as per Forest Plot	Calculation of Weighted Mean Dose per Study (I.U)	VitD Deficient(D) Sufficient(S) Insufficient(I)
Amestejani 2012 [39].	A, C	24	24	23 ± 3	55.4 ± 10.8	25 ± 4	15 ± 3	38%	<0.05	2	1600	31	496	D
Di Filippo 2015 [29]	C	22	22	57 ± 20	74 ± 26.8	46 ± 16	23 ± 15	51%	<0.001	3	1000	12.6	252	I
Javanbakht 2011 [40]	A,C	12	12	-	-	36 ± 4	23 ± 3	35%	0.004	2	1600	11.5	115	-
Samochocki 2013 [33]	A	20	20	19 ± 8	33 ± 14	45 ± 16	26 ± 11	43%	0.001	3	2000	29.3	469	D
Udompataikul 2015 [36]	C	12	12	43	63	18·± 9	8·± 9	56%	0.02	1	2000	15.6	312	D
								Weighted Mean Dose *				100%	**1600**	

Vitamin D status defined as a 25(OH)D—Deficiency <20 ng/mL (50 nmol/), Insufficiency 21–29 ng/mL (52. 5–72. 5 nmol/). Sufficiency >30 ng/mL (75 nmol/) [37]. * rounded value.

## Supplementary Materials

The following are available online at https://www.mdpi.com/2072-6643/11/8/1854/s1, Figure S1: Funnel Plot for meta-analysis of serum 25(OH)D levels in Atopic Dermatitis population compared with healthy controls (nmol/L), Figure S2: Funnel Plot of comparison of serum 25(OH)D levels in adult and paediatric Atopic Dermatitis population (nmol/L) versus their age-matched healthy controls, with sub-analysis by age group, Figure S3: Forest Plot for sub-analysis of vitamin D intervention trials in Atopic Dermatitis based on vitamin D dosage and time period, Table S1: Quality analysis of included observational studies using the Newcastle-Ottawa Scale, Table S2: Quality Analysis of included Interventional Studies using Cochrane Risk of Bias Scale, Table S3: Sensitivity Analysis of Meta-analysis of comparison of Serum 25(OH)D levels of AD vs. NonAD with each study exclusion, Table S4: Sensitivity analysis of Meta-analysis of comparison of Serum 25(OH)D levels of AD vs. Non-AD excluding studies from common regions and ethnicities, Table S5: Sensitivity Analysis of RCT Intervention trials of VitD supplementation in Atopic Dermatitis.
