# Supplementary material for: Vitamin D Deficiency and Effects of Vitamin D Supplementation on Disease Severity in Patients with Atopic Dermatitis: A Systematic Review and Meta-Analysis in Adults and Children
Source: Nutrients. 2019 Aug 9;11(8):1854. doi: 10.3390/nu11081854 (PMC6722944; doi:10.3390/nu11081854)
Supplement: Supplementary file 1 [file nutrients-11-01854-s001.pdf]

Vitamin D deficiency and effects of vitamin D supplementation on disease severity in patients with Atopic Dermatitis: a systematic review and meta-analysis in adults and children

Sonal R Hattangdi-Haridas, Susan A Lanham-New, Wilfred Hing Sang Wong, Marco Hok Kung Ho, Andrea L Darling

Contents

Systematic Review 2

Table S1: Quality analysis of included observational studies using the Newcastle-Ottawa Scale. 8

Table S2: Quality Analysis of included Interventional Studies using Cochrane Risk of Bias Scale 9

Table S3 : Sensitivity Analysis of Meta-analysis of comparison of Serum 25(OH)D levels of AD vs NonAD with each study exclusion 10

Table S4: Sensitivity analysis of Meta-analysis of comparison of Serum 25(OH)D levels of AD vs Non-AD excluding studies from common regions and ethnicities. 11

Table S5: Sensitivity Analysis of RCT Intervention trials of VitD supplementation in Atopic Dermatitis. 12

Figure S1: Funnel Plot for meta-analysis of serum 25(OH)D levels in Atopic Dermatitis population compared with healthy controls (nmol/L) 13

Figure S2: Funnel Plot of comparison of serum 25(OH)D levels in adult and paediatric Atopic Dermatitis population (nmol/L) versus their age-matched healthy controls, with sub-analysis by age group. 13

Figure S3: Forest Plot for sub-analysis of vitamin D intervention trials in Atopic Dermatitis based on vitamin D dosage and time period. 14

Supplementary References 15

Systematic Review

Serum 25(OH)D levels in AD vs. HC

Eight case-control studies (Tables 1-2) documented lower serum 25(OH)D levels in AD compared to HC: El Taieb et al. (2013)[1] (AD 5 ±2, HC 29 ± 2, p=0·001); Wang et al. (2014)[2] (11 ± 6, HC 14 ± 6 p<0·001); Cheon, et al. (2015)[3] (AD 23 ± 2HC 36± 3p<0·05); D'Auria et al. (2017)[4] (AD 19±7, HC 25±13 p= 0·04); Sharma et al. (2017)[5] (AD 12 ± 3,HC 21 ± 3 p=0·001); Su et al. (2017)[6](AD 16±7, HC 20±10 p=0·07); Noh et al. (2014)[7] (AD 10 ± 1, HC 11 ±1 p=0.001) and Han et al. (2015)[8](AD 12 ± 5, HC14 ± 6 p >0·05). Six of these studies evaluated paediatric groups, and were from Egypt,[1] Hong Kong,[2] Korea,[3] Italy,[4] India,[5] and Turkey.[6] In terms of methodology, two case-control studies, which covered both adult and child AD cases from South Korea,[7,8] studied winter 25(OH)D concentration. Four case-control studies excluded participants on VitD therapy or who had taken VitD within 2 to 6 months prior to the start of study.[1,4,5,8]

Wang et al. (2014)[2] found statistically significantly higher rates of vitamin D deficiency <25nm/L (10ng/ml) in Paediatric AD (48%) compared to same age HC (27%). They also found during regression analysis that serum 25(OH)D was a predictor of disease severity. Cheon et al. (2015)[3] and Sharma et al. (2017)[5] both documented statistically significantly lower serum 25(OH)D levels in the Paediatric AD population compared with Paediatric HC by 9 -11 ng/ml. Finally, Han et al. (2015)[8] reported statistically significantly lower 25(OH)D in AD patients compared with HC in children, albeit only by 3 nmol/l, but found no statistically significant difference in adults.

Relationship of 25(OH)D levels to AD disease severity

Note that no trials using EASI were found to be suitable for inclusion in the systematic review or meta-analysis. Five studies found that serum 25(OH)D in AD had an inverse association

with SCORAD score or Eczema involvement.[1-3,5,7] Four Paediatric studies reported a statistically significant negative relationship between 25(OH)D levels and SCORAD.[1,5,6,9] Specifically, Su et al. (2017)[6] reported lower serum 25(OH)D levels in moderate and severe AD compared to mild AD. Sharma et al.(2017)[5] documented serum 25(OH)D levels by severity, with more severe AD cases having statistically significantly lower 25(OH)D concentrations: mild 33 ± 6nmol/l; moderate 32 ± 6 nm/l; severe 21 ± 3 nmol/l. Similarly, El Taieb et al. (2013)[1] showed statistically significantly higher 25(OH)D levels in mild (15 = 4 ng/ml) compared to moderate (6±3ng/ml) and severe AD (0·3 ± 0·1 ng/ml). However, one study reported no relationship between serum 25(OH)D levels and AD severity or LL-37 levels[8] and another reported no relationship between 25(OH)D levels and SCORAD.[4] Finally, one last study reported that serum 25(OH)D levels were inversely related to the amount of body surface area affected by Eczema but not with the actual SCORAD score.[7]

Vitamin D supplementation and SCORAD Index in AD cases

In terms of intervention trial results (Tables 3-4), five intervention studies [9-13] reported reduction in SCORAD index in the VitD supplemented group compared to placebo (Javanbakht et al. (2011)[12]: -13pts (p=0·004), Amestejani et al. (2012)[13]: -10pts (p<0·005), Samochocki et al (2013)[11]: -20 pts (p=0·001), Udompataikul et al. (2015)[9]:-10pts (p=0. ·02), Di Filippo et al. (2015)[10]: -24 pts (p=0·01)). Udompataikul et al. (2015)[9] found a statistically significant negative correlation between 25(OH)D levels and SCORAD score. However, they found no improvement in lichenification, and neither did the study by Samochocki et al. (2013)[11] (Table 3).

In terms of intervention trial characteristics, three studies[9,12,13] were randomized controlled trials of one month each. In terms of dose and population, two studies[12] [13] supplemented 1600IU/daily in a population of teenagers to adults while Udompataikul et al. (2015)[9]

supplemented with 2000IU to a Paediatric age group. Samochocki et al. (2013)[11] and Di Filippo et al. (2015)[10] were three month interventional trials conducted during the winter to control for dermally produced 25(OH)D. Samochocki et al. (2013)[11] studied an exclusively adult population while Di Filippo et al. (2015)[10] studied a pre-pubertal paediatric population.

Relationship between serum 25(OH)D and SCORAD (AD severity)

Tsotra et al. (2017)[14] subdivided the AD population into two groups, 1200IU/daily for SCORAD<40 and 2400IU/daily for SCORAD>40, finding a statistically significant reduction of SCORAD from 62(42-75) to 5(0-37) in the 2400IU group only. Two interventional trials, Di Filippo et al. (2015)[10] ($r=-0{\cdot}5$) ($p<0{\cdot}001$) and Albenali et al. (2016)[15](no r value reported in paper, $p<0{\cdot}001$), reported that increases in serum 25(OH)D levels correlated inversely with SCORAD score. Albenali et al. (2016),[15] a clinical evaluation study, divided AD patients into 2 groups: (1-12yrs.) 6000IU daily; (12-18 yrs.) 10000 IU daily and found a statistically significant improvement in AD severity by 42% after 2 months of VitD supplementation.[15]

Effect of VitD Supplementation on serum cytokines

One Paediatric study[10] found that serum cytokine Levels (IL-2, IL-4, IL-6, IFN-γ) were higher in the AD group than in HC after 3 months of supplementation, but no difference in TNF-α. The study showed a statistically significant reduction in SCORAD (-24points).[10]

Relationship between 25(OH)D and serum IgE, total eosinophil count and atopic sensitisation

Three observational studies that focused on Paediatric populations found an inverse correlation between serum 25(OH)D and Serum IgE concentration.[2,3,6] Su et al. (2017),[6] based in

Turkey, found a statistically significant inverse correlation between serum 25(OH)D and IgE concentration, but no relationship between serum Ig E concentration and SCORAD. A large study by Wang et al. (2014)[2] from Hong Kong found that lower 25(OH)D status was associated with higher IgE in both AD patients and non-allergic controls. One South Korean study by Noh et al. (2014),[7] that assessed both Paediatric and adult populations, found no association between serum IgE and 25(OH)D levels in either AD or Asthma patients. Cheon, et al. (2015)[3] found a weak inverse correlation between SCORAD and serum IgE concentrations (r=0·2, p=0·03). Finally, one adult vitamin D supplementation trial[11] documented a statistically significant reduction in serum IgE levels after supplementation for three months (post supplementation: 995±1681 IU/ml; pre-supplementation: 1147±1884 IU/ml).

Three studies reported an inverse correlation between serum 25(OH)D concentration and Total Eosinophil Count (TEC) in AD patients.[2,3,7] In addition, Cheon et al. (2015)[3] documented a moderately strong, positive correlation between SCORAD and TEC (r=0·40, P<0·001). Only one study[3] assessed 25(OH)D and atopic sensitisation and found that children with atopic sensitization had a lower 25(OH)D concentration than those without.

Relationship between 25(OH)D concentration and skin bacterial infections

Two studies found a relationship between serum 25(OH)D levels and secondary bacterial skin infections in AD patients. First, an intervention trial by Samochocki et al. (2013)[11] selected n 98 AD patients, of which n 58 had dermal bacterial infections. AD patients with lower serum 25(OH)D levels (21±12·0 ng/ml) had a higher frequency of these infections (53.6%) compared to AD patients with higher serum levels (27±15 ng/ml), 46% of which had a dermal infection (p=0·03).[11] Of note, vitamin D supplemented AD patients did not report any bacterial infections during the trial period.[11] Udompataikul et al. (2015)[9] monitored Staph Aureus

(S.Aureus) infestation on the skin at 0, 2 and 4 weeks of VitD supplementation and found S.Aureus skin colonization statistically significantly reduced from baseline to week 4 in the intervention group compared with the placebo group. Udompataikul et al. (2015)[9] also found a statistically significant reduction of erythema index oedema, excoriation and pruritus.

Cathelicidin (LL-37) or Human Cathelicidin Antimicrobial Protein (CAMP/ hCAP18) in AD and its relation to 25(OH)D concentration

Two studies assessed the relationship between serum 25(OH)D levels and cathelicidin levels.[8,15] Han et al. (2015)[8] studied an adult and paediatric population and Albenali et al. (2016)[15] studied a Paediatric population using a novel noninvasive technique to measure LL-37 levels in the stratum corneum. Han et al (2015)[8] found a statistically significant inverse correlation between serum 25(OH)D concentration and serum cathelicidin (r=-0·300, p=0·011). However, on sub-analysis the result remained statistically significant only for adults (r= -0·4, p=0·03).[8] Albenali et al. (2016)[15] found that serum 25(OH)D levels correlated with lesional cathelicidin LL37 levels (r=0·3 p=0·02). Albenali et al. (2016)[15] also found that LL-37 cathelicidin levels were statistically significantly reduced in severe AD compared to mild AD, and lower lesional cathelicidin levels were recorded in Eczema Herpeticum (ADEH) patients than in AD patients (0·4 ±0·5 µg/g n=35 vs 0·5± 0·6 µg/g, n=12 (p=0·46)).[15] This is of importance as evidence suggests a direct correlation between lower 25(OH)D levels, lower dermal cathelicidin levels and higher risk of infection.[16]

Conversely, Tsotra et al. (2017)[14] documented significantly higher serum cathelicidin levels in AD children: 61 (26-129) vs HC: 50 (0·2-94)(p=0·02) at baseline. Lesional and serum cathelicidin levels did not relate to AD severity at baseline or after supplementation.[14] Downregulation of serum cathelicidin was documented in both interventional groups after 2 months of supplementation: 1200IU in Mild and 2400IU in Severe Atopic dermatitis (Mild AD

group: Baseline cathelicidin 63(26-129) post-supplementation 54(21 -92); Severe AD Group: Baseline cathelicidin 61(31-98·0), post-supplementation 52 (22-93).[14] However in contrast, Hata et al. (2014)[17] found no change in lesional cathelicidin levels in an adult AD population after VitD supplementation of 4000IU for twenty-one days. The technique of Real-Time Quantitative Reverse Transcription PCR (qRT_PCR) was performed on a punch biopsy of lesional skin to determine cathelicidin levels in the latter two studies.[17] [14]

IL-31, topical steroid usage, adverse effects and vitamin D type

Only one study[3] assessed IL-31 concentration and found that IL-31 levels were unrelated to AD severity (r=0·05,p=0·51) SCORAD index (mild AD 6±2, moderate AD 6±2, severe AD 6±2) (r=0·1, p=0·51) or serum 25(OH) D levels (r=0·2, p=0·79). IL-31 levels were not significantly different in AD compared to HC (p=0·13). Only one VitD supplementation study assessed topical steroid usage and found a statistically significant, strong reduction in topical steroid usage in AD patients in the intervention group (66·8%) compared to the placebo group (37·5%).[12] Three studies stated there were no adverse effects of Vitamin D supplementation[9,11,12] while the other five studies did not mention whether there were any adverse effects from VitD supplementation or not.[10,13-15,17] Of the eight interventional trials studied in this review, five used cholecalciferol (vitamin D_3).[11-13,15,17] One trial[9] used ergocalciferol (Vitamin D_2), while the other two trials[10,14] did not specify the form of VitD used in their intervention group.

Table S1: Quality analysis of included observational studies using the Newcastle-Ottawa Scale.

Study	Criterion Scores		
	Selection	Comparability	Exposure/Outcome
El Taieb 2013	**	**	**
Noh 2014	**	**	**
Wang 2014	***	**	**
Cheon 2015	**	**	**
Han 2015	***	**	**
D'Auria 2017	***	**	**
Su 2017	****	**	**
Sharma 2017	**	**	**

Table S2: Quality Analysis of included Interventional Studies using Cochrane Risk of Bias Scale

	Random Sequence generation (Selection bias)	Allocation concealment (selection bias)	Blinding of participants and personnel (performance bias)	Blinding of outcome assessment (detection bias)	Incomplete outcome data (attrition bias)		Selective reporting (reporting bias)	Other bias
**Javanbakht, 2011**	+	+	+	+	+		+	
**Amestejani, 2012**	+	+	+	+	-		+	
**Samochocki, 2013**	NA	NA	+	-	+		+	
**Hata 2014**	+	+	+	+	+		+	
**Di Filippo, 2015**	NA	NA	-	-	+		+	
**Udompataikul, 2015**	+	+	+	+	+		+	
**Albenali 2016**	-	-	-	+	+		+	
**Tsotra 2017**	-	-	-	+	+		?	

	Legends
+	**Low risk**
-	**High Risk**
?	**Uncertain**
NA	**Not applicable as repeated measures study**

Table S3 : Sensitivity Analysis of Meta-analysis of comparison of Serum 25(OH)D levels of AD vs NonAD with each study exclusion

Study excluded	Result
Cheon 2015[3]	-12 [95%CI -24 to 0·7] I^2 = 99% (P = 0·06)
D'Auria 2017[4]	-14 [95%CI -26 to -1] I^2 = 99% (P = 0·03)
Di Filippo[10] 2015	-16 [95%CI -28 to -3] I^2 = 99% (P = 0·01)
El Taieb 2013[1]	-9 [95%CI -17 to -1] I^2 = 98% (P = 0·02)
Han 2015[8]	-15 [95%CI -28 to -2] I^2 = 99% (P = 0·02)
Hata 2014[17]	-15 [95%CI -27 to -2] I^2 = 99% (P = 0·02)
Noh 2014[7]	-15 [95%CI -29 to -1] I^2 = 99% (P = 0·04)
Samochocki 2013[11]	-15 [95%CI -27 to -3] I^2 = 99% (P = 0·02)
Sharma 2017[5]	-13 [95%CI -26 to 0·4] I^2 = 99% (P = 0·06)
Su 2017[6]	-14 [95%CI -27 to -2] I^2 = 99% (P = 0·03)
Wang 2014[2]	-14 [95%CI -29 to -0·2] I^2 = 99% (P = 0·05)

Table S4: Sensitivity analysis of Meta-analysis of comparison of Serum 25(OH)D levels of AD vs Non-AD excluding studies from common regions and ethnicities.

**Study Excluded**	**Result**
South Korean studies [3,7,8]	-14 [95%CI -32 to 5] I^2 = 99% (P = 0·14)
Italian studies [4,10]	-16 [95%CI -29 to -3] I^2 =100% (P = 0·02).

Table S5: Sensitivity Analysis of RCT Intervention trials of VitD supplementation in Atopic Dermatitis.

**Study Excluded**	**Result**
Amestejani[13]	-13 [95%CI -15 to -10] I^2 = 0% (P <0·00001)
Javanbakht[12]	-10 [95%CI -12 to -8] I^2 = 0% (P <0·00001)
Udompataikul[9]	-11 [95%CI -14 to -8] I^2 = 65% (P <0·00001)

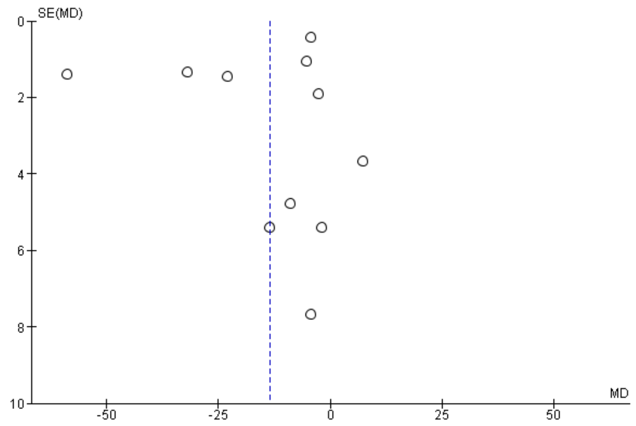

Figure S1: Funnel Plot for meta-analysis of serum 25(OH)D levels in Atopic Dermatitis population compared with healthy controls (nmol/L)

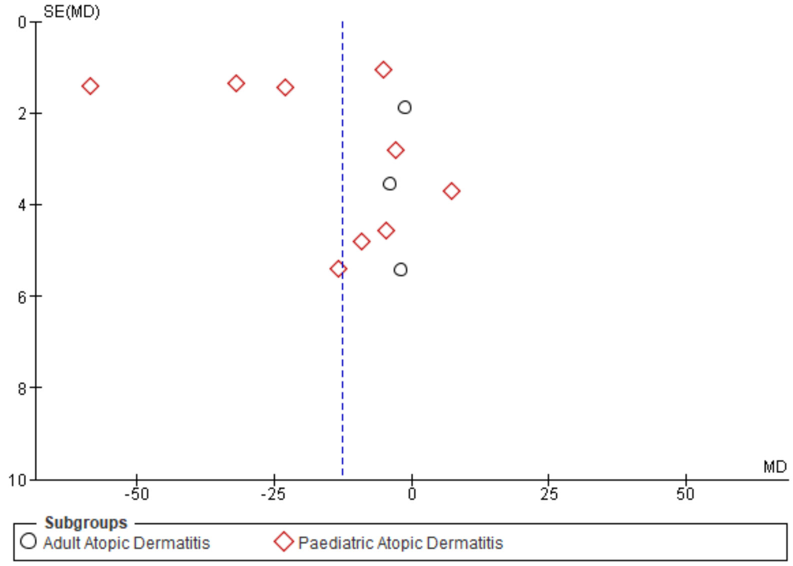

Figure S2: Funnel Plot of comparison of serum 25(OH)D levels in adult and paediatric Atopic Dermatitis population (nmol/L) versus their age-matched healthy controls, with sub-analysis by age group.

Study or Subgroup	post-VitD SCORAD Mean	post-VitD SCORAD SD	post-VitD SCORAD Total	Baseline SCORAD Mean	Baseline SCORAD SD	Baseline SCORAD Total	Weight	Mean Difference IV, Random, 95% CI
**1.5.1 Dosage VitD 1000IU Daily 3months**								
Di Filippo 2015	23	15	22	46	16	22	10.9%	-23.00 [-32.16, -13.84]
**Subtotal (95% CI)**			**22**			**22**	**10.9%**	**-23.00 [-32.16, -13.84]**
Heterogeneity: Not applicable								
Test for overall effect: Z = 4.92 (P < 0.00001)								
**1.5.2 Dosage Vitamin D1600IU Daily 2 months**								
Javanbakht 2011	23	3	12	36	4	12	29.6%	-13.00 [-15.83, -10.17]
Amestejani 2012	15	3	24	25	4	24	32.5%	-10.00 [-12.00, -8.00]
**Subtotal (95% CI)**			**36**			**36**	**62.1%**	**-11.33 [-14.25, -8.41]**
Heterogeneity: Tau² = 2.94; Chi² = 2.88, df = 1 (P = 0.09); I² = 65%								
Test for overall effect: Z = 7.60 (P < 0.00001)								
**1.5.3 Dosage Vitamin D 2000IU Daily 3 months**								
Samochocki 2013	26	11	20	45	16	20	12.1%	-19.00 [-27.51, -10.49]
**Subtotal (95% CI)**			**20**			**20**	**12.1%**	**-19.00 [-27.51, -10.49]**
Heterogeneity: Not applicable								
Test for overall effect: Z = 4.38 (P < 0.0001)								
**1.5.4 Dosage Vitamin D 2000IU Daily 1 month**								
Udompataikul 2015	8	9	12	18	9	12	14.9%	-10.00 [-17.20, -2.80]
**Subtotal (95% CI)**			**12**			**12**	**14.9%**	**-10.00 [-17.20, -2.80]**
Heterogeneity: Not applicable								
Test for overall effect: Z = 2.72 (P = 0.006)								
**Total (95% CI)**			**90**			**90**	**100.0%**	**-13.39 [-17.01, -9.77]**
Heterogeneity: Tau² = 9.45; Chi² = 12.43, df = 4 (P = 0.01); I² = 68%								
Test for overall effect: Z = 7.25 (P < 0.00001)								
Test for subgroup differences: Chi² = 8.35, df = 3 (P = 0.04), I² = 64.1%								

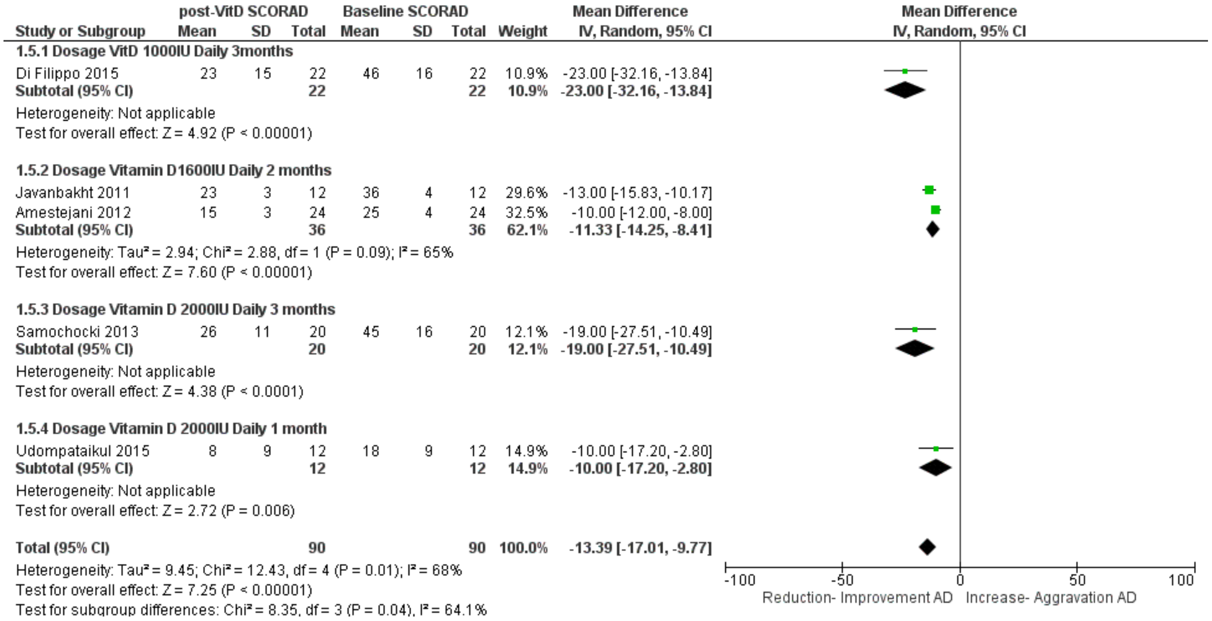

Figure S3: Forest Plot for sub-analysis of vitamin D intervention trials in Atopic Dermatitis based on vitamin D dosage and time period.

Supplementary References

1. El Taieb, M.A.; Fayed, H.M.; Aly, S.S.; Ibrahim, A.K. Assessment of serum 25-hydroxyvitamin d levels in children with atopic dermatitis: correlation with SCORAD index. *Dermatitis* **2013**, *24*, 296-301.
2. Wang, S.S.; Hon, K.L.; Kong, A.P.; Pong, H.N.; Wong, G.W.; Leung, T.F. Vitamin D deficiency is associated with diagnosis and severity of childhood atopic dermatitis. *Pediatr Allergy Immunol* **2014**, *25*, 30-35.
3. Cheon, B.R.; Shin, J.E.; Kim, Y.J.; Shim, J.W.; Kim, D.S.; Jung, H.L.; Park, M.S.; Shim, J.Y. Relationship between serum 25-hydroxyvitamin D and interleukin-31 levels, and the severity of atopic dermatitis in children. *Korean J Pediatr* **2015**, *58*, 96-101.
4. D'Auria, E.; Barberi, S.; Cerri, A.; Boccardi, D.; Turati, F.; Sortino, S.; Banderali, G.; Ciprandi, G. Vitamin D status and body mass index in children with atopic dermatitis: A pilot study in Italian children. *Immunol Lett* **2017**, *181*, 31-35.
5. Sharma, S.; Kaur, T.; Malhotra, S.K.; Rai, J.; Chaudhari, S. Correlation of Vitamin D3 Levels and SCORAD Index in Atopic Dermatits: A Case Control Study. *J Clin Diagn Res* **2017**, *11*, WC01-WC03.
6. Su, O.; Bahali, A.G.; Demir, A.D.; Ozkaya, D.B.; Uzuner, S.; Dizman, D.; Onsun, N. The relationship between severity of disease and vitamin D levels in children with atopic dermatitis. *Postepy Dermatol Alergol* **2017**, *34*, 224-227.
7. Noh, S.; Park, C.O.; Bae, J.M.; Lee, J.; Shin, J.U.; Hong, C.S.; Lee, K.H. Lower vitamin D status is closely correlated with eczema of the head and neck. *J Allergy Clin Immunol* **2014**, *133*, 1767-1770.e1766.
8. Han, T.Y.; Kong, T.S.; Kim, M.H.; Chae, J.D.; Lee, J.H.; Son, S.J. Vitamin D Status and Its Association with the SCORAD Score and Serum LL-37 Level in Korean Adults and Children with Atopic Dermatitis. *Ann Dermatol* **2015**, *27*, 10-14.
9. Udompataikul, M.; Huajai, S.; Chalermchai, T.; Taweechotipatr, M.; Kamanamool, N. The effects of oral vitamin D supplement on atopic dermatitis: A clinical trial with staphylococcus aureus colonization determination. *J Med Assoc Thai* **2015**, *98*, S23-S30.
10. Di Filippo, P.; Scaparrotta, A.; Rapino, D.; Cingolani, A.; Attanasi, M.; Petrosino, M.I.; Chuang, K.; Di Pillo, S.; Chiarelli, F. Vitamin D supplementation modulates the immune system and improves atopic dermatitis in children. *Int Arch Allergy Immunol* **2015**, *166*, 91-96.
11. Samochocki, Z.; Bogaczewicz, J.; Jeziorkowska, R.; Sysa-Jedrzejowska, A.; Glinska, O.; Karczmarewicz, E.; McCauliffe, D.P.; Wozniacka, A. Vitamin D effects in atopic dermatitis. *J Am Acad Dermatol* **2013**, *69*, 238-244.
12. Javanbakht, M.; Keshavarz, S.; Djalali, M.; Siassi, F.; Eshraghian, M.; Firooz, A.; Seirafi, H.; Ehsani, A.; Chamari, M.; Mirshafiey, A. Randomized controlled trial using vitamins E and D supplementation in atopic dermatitis. *J Dermatolog Treat*, **2011**; Vol. 22, pp 144-150.
13. Amestejani, M.; Salehi, B.; Vasigh, M.; Sobhkhiz, A.; Karami, M.; Alinia, H.; Kamrava, S.; Shamspour, N.; Ghalehbaghi, B.; Behzadi, A. Vitamin D supplementation in the treatment of atopic dermatitis: a clinical trial study. *J Drugs Dermatol* **2012**, *11*, 327-330.
14. Tsotra, K.; Garoufi, A.; Kossiva, L.; Gourgiotis, D.; Tsoukatou, T.; Katsantoni, E.; Stavropoulos, P. The impact of vitamin D supplementation on serum cathelicidin levels and the clinical course of atopic dermatitis in children. *Minerva Pediatr* **2017**, 10.23736/s0026-4946.17.04910-6.
15. Albenali, L.H.; Danby, S.; Moustafa, M.; Brown, K.; Chittock, J.; Shackley, F.; Cork, M.J. Vitamin D and antimicrobial peptide levels in patients with atopic dermatitis and atopic dermatitis complicated by eczema herpeticum: A pilot study. *J Allergy Clin Immunol* **2016**, *138*, 1715-1719.e1714.
16. Leaf, D.E.; Croy, H.E.; Abrahams, S.J.; Raed, A.; Waikar, S.S. Cathelicidin antimicrobial protein, vitamin D, and risk of death in critically ill patients. *Critical Care* **2015**, *19*, 80.

17. Hata, T.; Audish, D.; Kotol, P.; Coda, A.; Kabigting, F.; Miller, J.; Alexandrescu, D.; Boguniewicz, M.; Taylor, P.; Aertker, L., et al. A randomized controlled double-blind investigation of the effects of vitamin D dietary supplementation in subjects with atopic dermatitis. *J Eur Acad Dermatol Venereol:* 2014; Vol. 28, pp 781-789.
